# 4484 The Entrepreneurship for Biomedicine (E4B) Training Program

**DOI:** 10.1017/cts.2020.225

**Published:** 2020-07-29

**Authors:** Jane M. Garbutt, Joseph Grailer, Lillie Levin, Jessica Mozersky, Antes Schulke, Michael Kinch, Emre Toker

**Affiliations:** 1 Washington University in St. Louis, Institute Of Clinical and Translational Sciences

## Abstract

OBJECTIVES/GOALS: Regardless of their career choices, today’s biomedical researchers need to blend great science with core skills ininnovation and entrepreneurship (I&E). The objective of this NIH-funded education program was to develop and test a pragmatic training program to teach relevant I&E skills. METHODS/STUDY POPULATION: We used a modified Delphi approach to identify 15 relevant competencies for I&E and the essential topics to include in the program. Learner interviews identified preferences for online training programs (short, high-quality audio-visual content, ability to self-navigate, peer and instructor interactions). The inaugural program included 7 short, online courses that addressed how to identify and validate opportunities for innovation, sell your innovation to diverse audiences, assess its ethical consequences, work in teams, and develop resilience as an innovator. It also included mentor support, a team-based capstone project, and an optional in-person boot camp. RESULTS/ANTICIPATED RESULTS: 51 students enrolled and 41 participants from 9 institutions completed the program, including pre- and post-doctoral students and junior faculty. They organized into 10 teams to complete the capstone project, with 6 teams pitching their innovation to fellow students and mentors at the boot camp. Students rated satisfaction with courses highly overall, with 79% stating they would be disappointed if the program was no longer available. Preliminary results suggest participants increased their knowledge about and ability to perform tasks taught throughout the program. Suggestions for improvement included providing more practical advice and real-world examples to complement educational videos. DISCUSSION/SIGNIFICANCE OF IMPACT: The inaugural E4B program was well received and effective in increasing I&E skills. Improvements will include increased opportunity for mentor interactions and for advanced entrepreneurial training. The program is open for biomedical research trainees from all institutions with a CTSA award.

